# Supplementary material for: Profit shifting from Nigeria to Europe: The impact on human rights
Source: PLOS Glob Public Health. 2025 Mar 19;5(3):e0004218. doi: 10.1371/journal.pgph.0004218 (PMC11957553; doi:10.1371/journal.pgph.0004218)
Supplement: Attachment — Submitted filename: Response to Reviewers.docx [file pgph.0004218.s001.docx]

Reviewers' comments:

Reviewer's Responses to Questions

**Comments to the Author**

1. Does this manuscript meet PLOS Global Public Health’s [publication criteria](https://eur01.safelinks.protection.outlook.com/?url=https%3A%2F%2Fjournals.plos.org%2Fglobalpublichealth%2Fs%2Fcriteria-for-publication&data=05%7C02%7Crmep1%40st-andrews.ac.uk%7C53f365287d5e4891b7f408dc9c6a6656%7Cf85626cb0da849d3aa5864ef678ef01a%7C0%7C0%7C638557228007487235%7CUnknown%7CTWFpbGZsb3d8eyJWIjoiMC4wLjAwMDAiLCJQIjoiV2luMzIiLCJBTiI6Ik1haWwiLCJXVCI6Mn0%3D%7C0%7C%7C%7C&sdata=5JZJY0ocrOpY2k%2BXB3ZhFHFQ5aOG7jPv%2FcQ1U1jOb6I%3D&reserved=0)? Is the manuscript technically sound, and do the data support the conclusions? The manuscript must describe methodologically and ethically rigorous research with conclusions that are appropriately drawn based on the data presented.

Reviewer #1: No

**Authors’ response: We trust that the major revisions made to the manuscript and responses herewith ensure that Reviewer 1 now assesses the manuscript as meeting PLOS Global Public Health’s publication criteria.**

Reviewer #2: Yes

2. Has the statistical analysis been performed appropriately and rigorously?

Reviewer #1: I don't know

**Authors’ response: We have expanded the methodology to explain the GRADE econometric model in more detail with underlying formula. We have provided further information on Wier and Zucman’s latest data assessment of profits shifted as available on the Missing Profits website and for download.**

Reviewer #2: Yes

3. Have the authors made all data underlying the findings in their manuscript fully available (please refer to the Data Availability Statement at the start of the manuscript PDF file)?

The [PLOS Data policy](https://eur01.safelinks.protection.outlook.com/?url=https%3A%2F%2Fjournals.plos.org%2Fglobalpublichealth%2Fs%2Fdata-availability&data=05%7C02%7Crmep1%40st-andrews.ac.uk%7C53f365287d5e4891b7f408dc9c6a6656%7Cf85626cb0da849d3aa5864ef678ef01a%7C0%7C0%7C638557228007498821%7CUnknown%7CTWFpbGZsb3d8eyJWIjoiMC4wLjAwMDAiLCJQIjoiV2luMzIiLCJBTiI6Ik1haWwiLCJXVCI6Mn0%3D%7C0%7C%7C%7C&sdata=rEiItWayoyrwy9VILK%2FRQWX3BIzmwyShXgIBq%2BU4Bx4%3D&reserved=0) requires authors to make all data underlying the findings described in their manuscript fully available without restriction, with rare exception. The data should be provided as part of the manuscript or its supporting information, or deposited to a public repository. For example, in addition to summary statistics, the data points behind means, medians and variance measures should be available. If there are restrictions on publicly sharing data—e.g. participant privacy or use of data from a third party—those must be specified.

Reviewer #1: No

**Authors’ response: The expanded methodology makes our method clearer and the source of data and the GRADE model, both of which are available for download and use online.**

Reviewer #2: Yes

4. Is the manuscript presented in an intelligible fashion and written in standard English?

PLOS Global Public Health does not copyedit accepted manuscripts, so the language in submitted articles must be clear, correct, and unambiguous. Any typographical or grammatical errors should be corrected at revision, so please note any specific errors here.

Reviewer #1: No

**Authors’ response: We trust that the major revisions made to the manuscript and responses herewith ensure that Reviewer 1 now assesses the manuscript as meeting PLOS Global Public Health’s manuscript presentation guidelines.**

Reviewer #2: Yes

5. Review Comments to the Author

Please use the space provided to explain your answers to the questions above. You may also include additional comments for the author, including concerns about dual publication, research ethics, or publication ethics. (Please upload your review as an attachment if it exceeds 20,000 characters)

Reviewer #1: Thanks for your manuscript entitled European tax havens and their impact on fundamental rights in Nigeria and Europe.

The study aimed to assess the impact of shifted profits from Nigeria to all tax havens, including European ones, on fundamental rights and governance in Nigeria.

The authors have embarked on a crucial piece of work. The manuscript is not only fascinating but also a significant contribution to the field. The main missing link is the methodology. The authors have critiqued two methods to measure financial flows, but they have not described these methods or the methods they used to generate the results.

Consider revising the title to better reflect the purpose of the manuscript.
The manuscript assesses the impact of illicit financial flows from Nigeria to Europe on rights in Nigeria and Europe.

A comprehensive and clear description of the methodology used will significantly benefit the manuscript. This will provide readers with a better understanding of the research process and the validity of the results. Formula or model specifications and a description of the variables will also be useful.

Indicate the specific period that your research is looking at.

Included dates for profits shifted data (2019) and for estimated tax rate on shifted profits in European tax havens (2018).

Indicate how you calculated the profits and figured out that the cooperations shifted profits. One would expect international corporations to externalise their earnings, which may not be illegal. Maybe refrain from using the term "all tax havens" unless you know all the tax havens.

Describe what you mean by a tax haven and indicate a list of the leading tax havens for corporations working in Nigeria.

Indicate why the "profit shifting" affected sanitation, clean water and school attendance investments. If you have trend data on the trajectory of the investments in sanitation, water and school enrolment, you may show that "profit shifting" impacted this trajectory.

Minor issues
Please check the manuscript for grammar.
The financial disclosure should include the grant numbers
The data availability should include the specific data you used in the URL.
The total tax revenues in Table 2 do not add

In table 1, please indicate the source of the information in the table.
The limitations section essentially discusses the research without presenting the limitations. Describe the main limitations of the research, such as the lack of knowledge that if Nigeria did not lose resources from profit shifting, the resources would be invested correctly in sanitation, water, education, etc.

**Thank you, Reviewer 1, for your detailed feedback, especially on the methodology. We have checked the manuscript for grammatical errors and, as suggested, changed the title of the paper to better reflect the research purpose.**

**We have expanded the methodology so that the methods behind the GRADE econometric model are explained in more detail with the formula. This model is available online, and published descriptions of the model are cited. We briefly describe the two key methods for assessing profit shifting or profit misalignment and use the data produced by Wier and Zucman on profits shifted. This is available for download on the Missing Profits website and we have provided citations including URLs for this. We have used the most up-to-date figures.**

**The explanation of the methodology Wier and Zucman use to arrive at the revenue losses and gains for Nigeria and European tax havens has been further developed from the first draft of the manuscript, including their definition of tax havens and its limitations. The tax haven list, and the difference in dates for profits shifted data and the tax rate on shifted profits by European tax havens are the latest data from Wier and Zucman. We also are careful to not state that profit shifting is ‘illegal’.**

**We have clarified how we use Wier and Zucman’s profit-shifting results with the GRADE model to determine the impact on specific human rights. The expanded methodology also addresses the point about “lack of knowledge that if Nigeria did not lose resources from profit shifting, the resources would be invested correctly in sanitation, water, education, etc”. Although it is impossible to determine how a country in future would choose to spend resources it does not lose to profit shifting, the GRADE model—described in the methodology—uses four decades of historical data to analyse the relationship between government revenue, governance, and rights outcomes to estimate the impact of additional government revenue on citizens’ economic and social rights. That is, the model is designed to consider historic government allocations coupled with the impact of governance, so it is not assumed that Nigeria would invest all resources “correctly” or efficiently, or that all revenue would be unrealistically allocated to one sector.**

**On the minor issues, in terms of funding, 1) Scottish Funding Council International Science Partnership Fund 2023-24 number is SFC /AN/21/2023, and 2) The Professor Sonia Buist Global Child Health Research Fund is an alumni so there is no grant number.**

**Further, we have added a note under Table 3 to explain the differences (due to rounding). Sources have been added for the information in Table 1 (from the GRADE database). As is explained in the methodology, we use in GRADE the following data sources:**

- **GRADE: Government Revenue and Development Estimations (GRADE) (version 3.12.2), updated annually**
- **Government Revenue Central government revenue from the most recent version of the Government Revenue Database (GRD). All GRD data are presented as a percentage of GDP and are converted into GDP per capita using GDP per capita in constant 2015 USD from the World Development Indicators. GRD is typically updated annually.**
- **Governance: six Worldwide Governance Indicators (WGIs) (updated annually): Control of Corruption, Government Effectiveness, Voice and Accountability, Political Stability, Rule of Law, and Regulatory Quality (Kaufmann and Kraay, 2023). Each WGI is a composite score based on multiple surveys, with countries ranked on each indicator relative to the global average, which is set at zero, with values ranging from −2.5 +2.5.**
- **SDG/Human rights indicators: World Bank World Development Indicators (WDI) database, which is updated twice each year in April and September are used for the percentage of the population who access basic and safe water and sanitation, the percentage of the population who use clean fuels for cooking and who have access to electricity, the percentage of children aged less than five years who survive, and the percentage of women who survive childbirth. The United Nations Educational, Scientific, and Cultural Organization (UNESCO) Institute for Statistics (UIS) and the Global Education Monitoring Report recently adopted a Bayesian hierarchical cohort-based model to estimate out-of-school rates across all countries using data from administrative records and household surveys. The out-of-school rate (SDG indicator 4.1.4) is defined as the “percentage of children and young people within the official age range for a specific education level who are not enrolled in primary, lower and upper secondary education”. GRADE employs these estimates to model the impact of government revenue and governance on school attendance. UNESCO data are typically updated annually in September.**

Reviewer #2: This is an important research that examines a fundamental problem being faced by Nigeria in securing economic and social rights for its citizens. The authors have succeeded in offering very innovative arguments and analysis supported by credible data to link the sifting of profits to the loss of capability of the Nigerian state to secure many social determinants of health for their citizens. The aspect dealing with the presenation and analysis of the data was well done. However the bit you struggle is where you make normative arguments and discuss the relevant international human rights instruments and what they mean for the arguments of your paper. In particular, note the following more detailed comments:

• The conflation of Economic and Social Rights (ESR) with fundamental rights on paragraph 47 of the paper is misleading. In the literature, and under international human rights normativity, fundamental rights are associated with civil and political rights for which the principal treaty instrument is the ICCPR. On the other hand, the principal treaty instrument for ESRs is the ICESCR. These categories of rights do not carry the same impetus as civil and political rights. As such they cannot be conflated.

**Authors’ response: Thank you for this helpful clarification. We have removed the word ‘fundamental’ from our description of economic, social and cultural rights, and further expounded on the relationship between economic and social rights and development, as agreed in the UN Agenda 2030.**

• Para 51 - The extraterritorial enforcement of human rights by states is a questionable proposition given the obligation of state parties to treaties is to safeguard human rights commitments within their territory. You will need to develop your argument here further.

**Author’s response. Thank you for this observation. We have responded to this by expanding the discussion as well as adding further nuance to the background, the discussion of extraterritorial rights throughout the paper, and included a new table to show a selection of human rights committees' comments and observations on extraterritorial obligations for domestic tax policy.**

• The link which you make between the increase or decrease in government revenue and human rights protection is a pivotal part of your paper that needs to be more robustly established.

**Authors’ response: Thank you for pointing this out. The section on tax and human rights in the background has been greatly expanded and divided to provide a brief overview of the relationship between SDGs, rights, tax and government revenue, and empirical evidence.**

• The table 1 data is interesting. However, is it possible that there is more than one explanation accounting for the poor situation of the underlying determinants of health discussed in your paper that go beyond increase or decrease in revenue?

**Authors’ response: Thank you for this observation. We have added nuance to the presentation of data in Table 1 in the section “Evidence of government revenue impacts on rights and governance”, explaining the observations made at the population level for increases in revenue as well as those household and individual determinants.**

• Para 80 – the state safeguards rights; not provide rights. **This has been corrected.**
• Para 84 – states are the “duty bearers”. **This has been corrected**
• The quoted text in paras 143-148 should be indented rather than left intext – it is too long to be an intext quotation. **This has been corrected.**
• Para 162 – You use the acronym IFF which has not been previously used or defined in your paper. **Thank you for the observation. This has been corrected with an explanation provided based on the UN’s definition in the relevant SDG 16.4 and indicator.**
• Consider changing the focus of your paper from fundamental rights to economic and social rights as those are the most appropriate category of rights to which your paper relates. As I mentioned before, fundamental rights are more associated with civil and political rights, a different species of rights. **Thank you this has been addressed.**
• Para 325 – check for grammar. **Thank you, the issues have been addressed.**

• The normative argument you make on extraterritorial obligations for human rights enforcement which you ground on the UDHR and EU founding treaty opens up a lot space for controversy. Does the UDHR indeed create such an obligation? Which specific articles of the UDHR? The EU treaty is a regional instrument and cannot thus be the basis for the claim in para 358.
• It is true that the UDHR is the basis of support for subsequent international human rights treaties such as the ICCPR and ICESCR, however it will be good to refer your reader to specific articles of the ICESCR where the obligations you mentioned in para 364 of your paper can be found.
• In para 365 you reference General Comment 24. It is not clear which specific treaty or treaty body this General Comment relates to. You need to specify. Also keep in mind that General Comments are soft law and do not create binding statements of law to that extent. However, to the extent that they are interpretive tools for treaties, they carry a weight of their own.

**Authors’ response: thank you for these helpful observations and clarifications. The section on ‘Extraterritorial human rights obligations for cross-border tax policy” has been revised to bring clarity to UDHR, EU founding treating and the ICESCR, and this is now in the “Background”. We have striven to add nuance to the paper rather than making the normative argument on extraterritorial obligations, which, as you say, opens up a space for controversy, but rather argue that these guide external actions. Further literature has been consulted to support the arguments. Further clarifications have also been made on General Comment 24. Additional information on specific articles from the ICESCR have also been added, and a new Table on select UN committee, and expert statements on extraterritorial human rights obligations and cross-border tax policy to show the “growing body of literature that shows that shows the scale of offshore revenue leakages from lower-income countries has implications for international tax and human rights law (Michelmore, 2024)”, as we write in the conclusion.**

6. PLOS authors have the option to publish the peer review history of their article ([what does this mean?](https://eur01.safelinks.protection.outlook.com/?url=https%3A%2F%2Fjournals.plos.org%2Fglobalpublichealth%2Fs%2Feditorial-and-peer-review-process%23loc-peer-review-history&data=05%7C02%7Crmep1%40st-andrews.ac.uk%7C53f365287d5e4891b7f408dc9c6a6656%7Cf85626cb0da849d3aa5864ef678ef01a%7C0%7C0%7C638557228007505896%7CUnknown%7CTWFpbGZsb3d8eyJWIjoiMC4wLjAwMDAiLCJQIjoiV2luMzIiLCJBTiI6Ik1haWwiLCJXVCI6Mn0%3D%7C0%7C%7C%7C&sdata=8Rh74rgHlx5nn8BI2h5tFgz3dvCIbsHae3bLQuj5IYk%3D&reserved=0)). If published, this will include your full peer review and any attached files.


**Do you want your identity to be public for this peer review?** If you choose “no”, your identity will remain anonymous but your review may still be made public.


For information about this choice, including consent withdrawal, please see our [Privacy Policy](https://eur01.safelinks.protection.outlook.com/?url=https%3A%2F%2Fwww.plos.org%2Fprivacy-policy&data=05%7C02%7Crmep1%40st-andrews.ac.uk%7C53f365287d5e4891b7f408dc9c6a6656%7Cf85626cb0da849d3aa5864ef678ef01a%7C0%7C0%7C638557228007513014%7CUnknown%7CTWFpbGZsb3d8eyJWIjoiMC4wLjAwMDAiLCJQIjoiV2luMzIiLCJBTiI6Ik1haWwiLCJXVCI6Mn0%3D%7C0%7C%7C%7C&sdata=3WtLfyupoGOAisaqJPf57T1k7slUviR0%2FtzczT8RNsU%3D&reserved=0).

Reviewer #1: No

Reviewer #2: **Yes:** Dr Uchechukwu Ngwaba

[NOTE: If reviewer comments were submitted as an attachment file, they will be attached to this email and accessible via the submission site. Please log into your account, locate the manuscript record, and check for the action link "View Attachments". If this link does not appear, there are no attachment files.]

While revising your submission, please upload your figure files to the Preflight Analysis and Conversion Engine (PACE) digital diagnostic tool, <https://pacev2.apexcovantage.com/>. PACE helps ensure that figures meet PLOS requirements. To use PACE, you must first register as a user. Registration is free. Then, login and navigate to the UPLOAD tab, where you will find detailed instructions on how to use the tool. If you encounter any issues or have any questions when using PACE, please email PLOS at [figures@plos.org](mailto:figures@plos.org). Please note that Supporting Information files do not need this step.
